# Experiences of Birth Attendants on Upward Obstetric Emergency Referrals in Low- and Middle-Income Countries: Protocol for a Scoping Review

**DOI:** 10.2196/64886

**Published:** 2025-04-10

**Authors:** Final Z Juqu, Olivia B Baloyi, Esther L Mbobnda Kapche, Wilma ten Ham-Baloyi, Geldine Chironda, Zamadonda Nokuthula Xulu-Kasaba

**Affiliations:** 1 School of Nursing and Public Health College of Health Sciences University of KwaZulu-Natal Durban South Africa; 2 Department of Nursing Science Faculty of Health Science Nelson Mandela University Port Elizabeth South Africa; 3 Seed Global Health St John of God University Mzuzu Malawi; 4 Department of Optometry College of Health Sciences University of KwaZulu-Natal Durban South Africa

**Keywords:** referral systems, upward referral, obstetric emergencies, traditional birth attendants, skilled birth attendants, low- and middle-income countries, birth attendants, obstetric, middle-income countries, scoping review protocol, pregnancy, neonatal deaths, deaths, obstetric care, health care, medical education, mortality, Africa, pregnant, women’s health

## Abstract

**Background:**

Every day, approximately 800 women die from pregnancy-related causes, alongside 2.6 million stillbirths and 2.8 million neonatal deaths annually. Inadequate referral by skilled birth attendants hinders timely access to necessary emergency obstetric care, challenging progress toward the maternal health Sustainable Development Goal (SDG) 3. The COVID-19 pandemic further disrupted care in low- and middle-income countries, forcing women to rely on traditional birth attendants, thereby affecting the referral system. It is crucial to understand the experiences of both skilled and traditional birth attendants regarding upward referrals in emergency obstetric care to identify barriers and facilitators within these systems in low- and middle-income countries.

**Objective:**

This study aims to map existing evidence on the experiences of skilled and traditional birth attendants regarding upward referral systems in emergency obstetric care within low- and middle-income countries.

**Methods:**

We will conduct a scoping review guided by the Joanna Briggs Institute’s methodological framework. Studies will be included if they report on experiences with upward referral in obstetrical emergencies. We will consider studies published in English and French from 2016 to July 2024. The literature search will be conducted in databases including PubMed, EBSCOhost (Academic Search Complete and CINAHL with full text), Scopus, Web of Science, and Google Scholar. Identified citations will be managed using EndNote version 21 (Clarivate Analytics) and Rayyan. Two independent reviewers will screen eligible studies and resolve disagreements through discussion with a third reviewer. Data will be extracted using a validated form and analyzed through content analysis, with findings presented narratively. This protocol aligns with the PRISMA-ScR (Preferred Reporting Items for Systematic Reviews and Meta-Analyses Extension for Scoping Reviews) guidelines. The review will offer a comprehensive narrative of upward referral systems in obstetrical emergencies, focusing on transitions from traditional birth attendants to health care facilities and from lower to higher levels of health care.

**Results:**

The preliminary search was completed in August 2024, and the database search will be conducted within the next 6 months. Findings will be disseminated through medical education conferences and publications.

**Conclusions:**

This review contributes a comprehensive narrative of upward referral systems in obstetrical emergencies, aiming to enhance understanding and improve transitions from traditional birth attendants to health care facilities and between different health care levels. It could significantly impact maternal and neonatal care by identifying the referral experiences of both skilled and traditional birth attendants. The insights may inform interventions that integrate traditional birth attendants into health care systems, potentially reducing maternal and neonatal mortality. The results will guide future research aimed at developing a model to improve upward referrals for obstetric emergencies in sub-Saharan Africa.

**Trial Registration:**

Open Science Framework 4HAVZ; https://osf.io/4havz

**International Registered Report Identifier (IRRID):**

PRR1-10.2196/64886

## Introduction

Pregnancy and childbirth can pose risks to both the mother and fetus [1], sometimes leading to life-threatening situations known as obstetric emergencies [2]. In such cases, specialized care is necessary and provided through a well-established referral system [3], typically an upward referral, which refers to the process by which health care providers at lower levels of the system seek assistance from specialized or better-equipped providers [4]. This system ensures a smooth transfer of patients between different levels of health care facilities, optimizes service delivery, and reduces the risk of complications [5] that can lead to mortality. Skilled birth attendants (SBAs) and traditional birth attendants (TBAs) are crucial in this process [6,7]. Alongside pregnant women, these frontline caregivers navigate the complexities of pregnancy and childbirth, often requiring swift intervention to ensure positive maternal and neonatal outcomes. However, the varying experiences of different stakeholders can negatively impact the obstetric referral process [8].

Every day, approximately 800 maternal deaths occur due to pregnancy-related causes [8], alongside 2.6 million stillbirths and 2.8 million neonatal deaths annually [9]. The global community is committed to improving health care quality to address these alarming statistics [8]. In line with Sustainable Development Goal (SDG) 3, which aims to ensure healthy lives and well-being for all, specific targets highlight the urgency of action. SDG 3, Target 1, aims to reduce the global maternal mortality ratio (MMR) to below 70 maternal deaths per 100,000 live births by 2030. Target 2 seeks to prevent the deaths of newborns and children younger than 5 years to fewer than 25 deaths per 1000 live births and reduce neonatal mortality to 12 deaths per 1000 live births [9]. However, challenges remain, especially in low- and middle-income countries (LMICs) [8], which are defined as countries with a gross national income per capita ranging from US $1086 to US $4255 [10]. Despite the United Nations’ recognition of the right to access the highest attainable standard of physical health, including the integration of referral systems [11], obstetric emergencies continue to pose significant threats to the health and lives of mothers and newborns worldwide [8]. TBAs historically played a pivotal role in maternal health care before the formalization of midwifery, but interest in their role has diminished over time [6,12]. Since the 2000s, the focus has shifted toward skilled birth attendance, sidelining TBAs in routine deliveries due to their ineffectiveness in reducing maternal mortality [7]. Nevertheless, a significant proportion of women in LMICs continue to seek TBA services, highlighting their enduring importance [13].

In contrast, SBAs are recognized as a key strategy for reducing high maternal mortality rates and are pivotal indicators of progress in maternal mortality reduction efforts [7]. Addressing high maternal mortality rates through referral systems requires a comprehensive understanding of the experiences of pregnant women, SBAs, and TBAs [7]. However, the current health care system operates in fragmented silos, hindering collaboration between different systems. This lack of integration undermines efforts to combat maternal mortality, despite some pregnant women preferring TBA practices over Western medicine [12].

Research based on the Three Delays Model by Thaddeus and Maine highlights challenges affecting the timeliness of obstetric referrals within health care facilities. These challenges occur at both patient and institutional levels, impacting the referral process [7]. They include difficulties in identifying and reaching health care facilities, often involving TBAs in the referral process, and obstacles in receiving adequate care, often linked to SBAs who are responsible for essential obstetric care during pregnancy [14].

To improve maternal health outcomes, it is crucial to explore and understand the experiences of women, TBAs, and SBAs. Understanding stakeholders’ perceptions and navigation of the referral process can help identify gaps in service delivery, such as limited access to emergency obstetric care facilities or communication challenges between health care providers and pregnant women. Addressing these challenges can enhance the efficiency and effectiveness of obstetric referral systems, ensuring timely access to needed care for women.

Although previous studies and reviews have examined referrals in obstetric emergencies [1-3,5,8,14] since the adoption of the SDGs, none have focused on the experiences of upward obstetric emergency referrals in LMICs for both SBAs and TBAs, advocating for integrated referral systems that can save the lives of mothers and babies. Therefore, this review aims to provide a comprehensive overview of the current state of birth attendants’ experiences with referral systems in obstetric emergencies in low-income countries.

## Methods

### Study Design

Using the steps of the Joanna Briggs Institute (JBI) Scoping Review Methods [14], the researchers will examine and map the available literature related to the experiences of birth attendants with upward obstetric emergency referrals in LMICs. This scoping review protocol has been developed and registered with the Open Science Framework. The protocol will be implemented, and the PRISMA-ScR (Preferred Reporting Items for Systematic Reviews and Meta-Analyses Extension for Scoping Reviews) guidelines will be used to guide the reporting.

### Main Objective and Review Questions

The main objective of this review is to examine available evidence regarding birth attendants’ experiences with upward referral systems in emergency obstetric care in sub-Saharan Africa (SSA) since the inception of the SDGs. Based on the objective of this review, the following research questions have been developed:

1. What are the publication characteristics of evidence on upward obstetric referrals?
2. What are the experiences of birth attendants regarding upward obstetric emergency referrals in LMICs?

The objective of this scoping review is part of the PhD study titled “Developing a Model to Enhance Effective Upward Referral of Obstetric Emergencies from Community Health Centres in Oliver Tambo District, Eastern Cape, South Africa: A Grounded Theory Inquiry.”

### Eligibility Criteria

The eligibility criteria for this review will be based on the JBI mnemonic for formulating systematic review questions, which describe the population, concept, and context of the study [14]. Textbox 1 summarizes the criteria.

**Eligibility criteria.**

**Inclusion criterion:**

- Population:
  - Skilled birth attendant: a professional health care worker who attends to pregnant women
  - Traditional birth attendant: an unskilled person who attends to pregnant women
- Concept:
  - Upward referral of women during labor and childbirth
  - Referral of women during pregnancy
  - Referral from traditional birth attendants to a health care facility
- Context:
  - Low- and middle-income countries
- Study designs:
  - Qualitative designs
  - Quantitative designs
  - Mixed methods designs
  - Conference proceedings abstracts
  - Gray literature
- Time period:
  - From January 2016 to July 2024

**Exclusion criterion:**

- Population:
  - Professional health care workers who do not attend to pregnant women
  - Unskilled individuals who do not attend to pregnant women
- Concept:
  - Referral of women outside the pregnancy and childbirth experience
  - Neonatal emergencies
- Context:
  - High-income countries
- Study designs:
  - Letters to the editor
  - Reviews
- Time period:
  - After July 2024

#### Population

The scoping review will include all relevant peer-reviewed and gray literature focused on upward referral systems in emergency obstetric care in LMICs. The population sample for the review will consist of birth attendants including SBAs and TBAs.

#### Concept

The concept will be guided by the following: Experiences, perceptions, and upward obstetric emergency referrals**.** According to the World Health Organization (WHO) [15], “referral can be defined as a process in which a health worker at one level of the health care system, having insufficient resources (eg, drugs, equipment, and skills) to manage a clinical condition, seeks the assistance of a better or differently resourced facility at the same or higher level to assist in or take over the management of the client’s case.” Furthermore, an obstetric emergency is defined as a complication or situation of a serious and often dangerous nature, developing suddenly and unexpectedly, and demanding immediate attention in order to save lives [8].

#### Context

The context of this review will focus on LMICs within emergency obstetric care settings.

### Types of Sources

This scoping review will include both experimental and quasi-experimental study designs such as before-and-after studies and interrupted time-series studies. In addition, analytical observational studies, including prospective and retrospective cohort studies, case-control studies, and analytical cross-sectional studies, will be considered for inclusion. Descriptive observational study designs, including case series, individual case reports, and descriptive cross-sectional studies will also be included. Qualitative studies focusing on qualitative data, including, but not limited to, designs such as phenomenology, grounded theory, ethnography, qualitative description, action research, and feminist research, will be considered for inclusion. Furthermore, text and opinion papers will also be considered in this scoping review.

### Search Strategy

An initial limited search in PubMed and ScienceDirect was undertaken to identify papers on the topic. The text words from the titles and abstracts of relevant papers, along with the index terms used to describe them, were used to develop a full search strategy (Table 1).

**Table 1:** Preliminary search strategy.

Date	Database	Search query	Results, n
January 29, 2024	PubMed	((“obstetric labor complications”[MeSH Terms] OR (“obstetric”[All Fields] AND “labor”[All Fields] AND “complications”[All Fields]) OR “obstetric labor complications”[All Fields]) AND (“referral and consultation”[MeSH Terms] OR (“referral”[All Fields] AND “consultation”[All Fields]) OR “referral and consultation”[All Fields] OR (“hospital”[All Fields] AND “referrals”[All Fields]) OR “hospital referrals”[All Fields]) AND (“developing countries”[MeSH Terms] OR (“developing”[All Fields] AND “countries”[All Fields]) OR “developing countries”[All Fields])) AND (2016/1/1:2024/1/31[pdat])	11
July 3, 2024	Science Direct	referral OR referral process OR referral pathway OR care pathway) AND obstetric labour complications AND Developing countries	712

Second, a systematic search will be conducted across 4 remaining electronic databases, namely EBSCOhost (including Academic Search Complete and CINAHL with full text), Scopus, Web of Science, and Google Scholar.

The search strategy, including all identified keywords and index terms, will be adapted for each included database or information source or both. Peer-reviewed journals will be reviewed for primary studies with a clear empirical base, using qualitative, quantitative, and mixed methods addressing the research question. Studies will be identified by searching literature from January 2016 to July 2024. In addition, papers will be searched through the “cited by” search feature as well as through citations included in the reference lists of included papers. The search terms will include referral system, LMICs, obstetric emergency, SBAs, and TBAs. Boolean operators (AND, OR) will be used to separate keywords, and Medical Subject Headings (MeSH) terms will be included during the search. The search syntax will be modified as needed. Reference lists of selected papers will also be searched for additional papers of interest. The services of an experienced subject librarian will be used to ensure that a robust search strategy is followed. The search strategy will be piloted to check the appropriateness of selected electronic databases and keywords. To compile all relevant evidence sources, identify, and remove duplicate records, the EndNote X21 reference manager will be used to import and manage eligible studies.

### Source of Evidence Selection

Following the search, all identified citations will be collated and uploaded into EndNote version 21, and then imported into the Ryann systematic review app, where duplicates will be removed. Following a pilot test, titles and abstracts will be screened independently by FZJ and ELMK for assessment against the inclusion criteria for the review. Potentially relevant sources will then be retrieved in full. The full text of selected citations will be assessed independently by FZJ and ELMK to determine whether they meet the inclusion criteria. Reasons for the exclusion of sources of evidence in the full text that do not meet the inclusion criteria will be recorded and reported in the scoping review. Any disagreements between the reviewers at each stage of the selection process will be resolved by OBB. The results of the search and study inclusion process will be fully reported in the final scoping review and presented in a PRISMA-ScR flow diagram [16]*.*

### Data Extraction

Data will be extracted from the papers included in the scoping review by FZJ and ELMK independently, using a data extraction tool developed by the reviewers. The data extracted will be informed by the formulated review questions and will include—in addition to paper details such as authors, year of publication, design, and setting—specific details about the participants or population, reasons for referral, challenges experienced, support identified related to the referral, and further recommendations for upward obstetric emergency referrals. Before data extraction, the tool will be pilot-tested independently by FZJ and ELMK. The draft data extraction tool will be modified and revised as necessary during the process of extracting data from each included evidence source. Any modifications will be documented in the scoping review. OBB will afterwards check that no relevant information has been omitted. The information to be extracted using this tool is detailed below (Textbox 2).

**Initial data extraction tool.**

**Criteria:**

- Authors or year of publication or both
- Research design
- Study setting
- Nature of population or sample
- Reasons for referral
- Challenges experienced related to the referral
- Support experience related to the referral
- Recommendations related to the referral

### Data Analysis

Knowledge and experiences of skilled and traditional BAs regarding referral in obstetric emergencies to combat maternal and child mortality rates will be extracted from this review. The data will be analyzed to develop a comprehensive model for effective referral in resource-constrained settings.

Data summarization and reporting will adopt a fundamental descriptive approach, using content analysis [17]. A narrative approach will present the findings from the included studies, using thematic content analysis to describe the themes that are relevant to experiences with referral systems in obstetric emergencies in low- and middle-income countries. In addition, any other emerging themes will be reported.

### Ethical Considerations

This study was approved by the university’s ethics committee for BioMedical Research (ethics approval BREC/00006633/2024).

## Results

As of August 2024, the preliminary search was completed, and the database search will be conducted within the next 6 months. Findings will be disseminated through medical education conferences and publications.

## Discussion

### Principal Findings

It is anticipated that this review will map out the different experiences of birth attendants regarding upward referral in obstetric emergencies. It is also anticipated that the experiences of SBAs are different from those of TBAs. These insights will enrich the body of knowledge related to referrals in LMICs. The findings have the potential to aid in improving care for mothers and newborns in several ways. First, the study will synthesize information on referral systems in obstetric emergencies within resource-constrained health care settings. Second, it will identify referrals from both SBAs and TBAs to health care facilities. This information could support the development of interventions that advocate for including TBAs in health care systems to reduce maternal and neonatal deaths. Third, the review’s findings will inform a future study aimed at developing a model to enhance effective upward referral of women with obstetric emergencies during labor in an SSA country.

Comparatively, researchers [1,2] have highlighted logistical challenges in emergency referrals, concentrating on gaps in primary care systems and delays in reaching tertiary facilities. This study, on the other hand, provides a more comprehensive understanding of the systemic and interpersonal dynamics influencing emergency referrals in LMICs. The dual focus on both TBAs and SBAs is unique, as it addresses an unexplored area in studies such as [7], which observed that traditional and formal care providers act as fragmented health structures, preventing collaboration. However, in LMICs, a significant proportion of women continue to seek TBA services, highlighting their enduring importance [13]. Therefore, the insights generated could serve as a foundation and guidance for future research aimed at developing a model to improve upward referrals for obstetric emergencies in SSA.

### Policy and Practical Implications

The scoping review aims to provide insights into the experiences of both SBAs and TBAs regarding upward referral systems in emergency obstetric care. It will be instrumental in identifying key gaps and challenges in the referral process.

By linking the anticipated results to the SDGs, particularly SDG 3 on good health and well-being, the review highlights the critical role of improving referral systems to reduce maternal and neonatal mortality. The review will inform policy makers about effective strategies to enhance referral processes, ensure timely access to emergency obstetric care, and better integrate TBAs into the health care system.

Health care providers are expected to benefit from the insights this scoping review will provide regarding training needs for effective referrals and collaboration between SBAs and TBAs. This will improve referral practices and ensure smoother transitions of care.

Furthermore, the review will emphasize the importance of community involvement in supporting and understanding the referral process. Recommendations for engaging communities through educational campaigns and support systems may strengthen the overall referral network.

### Potential Challenges and Limitations

The review includes only studies published in English and French, which may exclude relevant research in other languages and limit the comprehensiveness of the findings. Furthermore, it focuses on studies published and indexed in selected databases, potentially overlooking unpublished research.

### Conclusion

This review contributes a comprehensive narrative on upward referral systems in obstetrical emergencies, aiming to enhance understanding and improve transitions from TBAs to health care facilities and between different health care levels. It could significantly impact maternal and neonatal care by describing the referral experiences of skilled TBAs in obstetric emergencies. The insights may inform interventions that integrate TBAs into health care systems, potentially reducing maternal and neonatal mortality. The results will guide future research aimed at developing a model to improve upward referrals for obstetric emergencies in an SSA country.

## Abbreviations

JBI: Joanna Briggs Institute
LMIC: low- and middle-income countries
MeSH: Medical Subject Headings
MMR: maternal mortality ratio
PRISMA-ScR: Preferred Reporting Items for Systematic Reviews and Meta-Analyses Extension for Scoping Reviews
SBA: skilled birth attendant
SDG: Sustainable Development Goal
SSA: sub-Saharan Africa
TBA: traditional birth attendant
WHO: World Health Organization

## Data Availability

All data generated and analyzed will be included in the published scoping review article and will be available upon request.

## References

[1] Prathiba P, Niranjjan R, Maurya DK, Lakshminarayanan S (2020). Referral chain of patients with obstetric emergency from primary care to tertiary care: a gap analysis. J Family Med Prim Care.

[2] Leta M, Assefa N, Tefera M (2022). Obstetric emergencies and adverse maternal-perinatal outcomes in Ethiopia; a systematic review and meta-analysis. Front Glob Womens Health.

[3] Ximba SW, Baloyi OB, Ann Jarvis M (2021). Midwives' perceived role in up referral of high-risk pregnancies in primary healthcare settings, eThekwini district, South Africa. Health SA.

[4] (2020). Referral policy for South African health services and referral implementation guidelines. KnowledgeHub.

[5] Wanja T, Njoroge K, Osoro EN (2021). Factors influencing upward referral system of patients in Nairobi County. Int J Community Med Public Health.

[6] Jean-Baptiste M, Millien C, Pognon PR, Jean-Baptiste MC (2023). Reframing the three delays framework: factors influencing referrals to facilities by matrones in rural Haiti. BMJ Glob Health.

[7] Uny I, de Kok B, Fustukian S (2019). Weighing the options for delivery care in rural Malawi: community perceptions of a policy promoting exclusive skilled birth attendance and banning traditional birth attendants. Health Policy Plan.

[8] Nabulo H, Gottfredsdottir H, Joseph N, Kaye DK (2023). Experiences of referral with an obstetric emergency: voices of women admitted at Mbarara Regional Referral Hospital, South Western Uganda. BMC Pregnancy Childbirth.

[9] (2023). Trends in maternal mortality 2000 to 2020: estimates by WHO, UNICEF, UNFPA, World Bank Group and UNDESA/Population Division. World Health Organization.

[10] (2022). Low- and middle-income countries (LMIC) classification. World Bank.

[11] (2015). Sustainable development goals helpdesk. Food and Agriculture Organizations of the United Nations.

[12] (2016). World health statistics 2016: monitoring health for the SDGs, sustainable development goals. World Health Organization.

[13] Musie MR, Mulaudzi MF, Anokwuru R, Bhana-Pema V (2022). Recognise and acknowledge us: views of traditional birth attendants on collaboration with midwives for maternal health care services. Int J Reprod Med.

[14] Garces A, McClure EM, Espinoza L, Saleem S, Figueroa L, Bucher S, Goldenberg RL (2019). Traditional birth attendants and birth outcomes in low-middle income countries: a review. Semin Perinatol.

[15] Dzomeku VM, Mensah ABB, Nakua EK, Agbadi P, Okyere J, Kumah A, Munukpa J, Ofosu AA, Lockhart N, Lori JR (2023). Perspectives of healthcare workers on the challenges with obstetric referrals in rural communities in Ghana: a descriptive phenomenology study. BMJ Open.

[16] Joanna Briggs Institute (2024). Joanna Briggs Institute Reviewers Manual: 2024 edition.

[17] (2014). Referral. World Health Organization.

